# Prenatally Diagnosed 7q11.23 Copy Number Variations: A Retrospective Case Series

**DOI:** 10.1002/mgg3.70181

**Published:** 2026-01-06

**Authors:** Jiong Yan, Ziyang Liu, Song Yi, Nian Liu

**Affiliations:** ^1^ Department of Administration Office, Maternal and Child Health Hospital of Hubei Province, Tongji Medical College Huazhong University of Science and Technology Wuhan China; ^2^ College of Agronomy and Biotechnology Southwest University Chongqing China; ^3^ Medical Genetic Center, Maternal and Child Health Hospital of Hubei Province, Tongji Medical College Huazhong University of Science and Technology Wuhan China

**Keywords:** 7q11.23 microdeletion, genetic inheritance, prenatal counseling, prenatal ultrasound, SNP array, Williams‐Beuren syndrome

## Abstract

**Background:**

Williams‐Beuren syndrome (WBS; OMIM #194050), caused by 7q11.23 deletions, is well‐characterized postnatally, but prenatal manifestations remain poorly defined. This study aims to delineate the prenatal phenotypes, inheritance patterns, and outcomes of 7q11.23 copy number variations (CNVs).

**Methods:**

A retrospective study of 20 prenatal cases with 7q11.23 CNVs diagnosed by SNP array or CNV sequencing (CNV‐seq) was conducted. Clinical data, including ultrasound findings, genetic results, and pregnancy outcomes, were analyzed.

**Results:**

Classic 7q11.23 deletions (1.42 Mb median size) were associated with ultrasound anomalies in 100% of cases (11/11), predominantly cardiovascular defects (36.4%, 4/11) and growth restriction (18.2%, 2/11). While 7q11.23 duplications (1.42–3.03 Mb) were associated with anomalies in 50% of cases (3/6), including cleft palate and ventriculomegaly. Inheritance pattern analysis revealed 50% of deletions (6/12) and 42.9% of duplications (3/7) were inherited, either from phenotypically normal or abnormal parents. Termination of pregnancy (TOP) occurred in 76.5% (13/17) of ongoing pregnancies, primarily for de novo CNVs. Four live births involved inherited CNVs.

**Conclusion:**

7q11.23 CNVs exhibit significant prenatal phenotypic variability and inheritance heterogeneity. Advanced genomic testing and inheritance pattern analysis are critical for accurate diagnosis and counseling.

## Graphical abstract

Prenatal 7q11.23 CNVs exhibit 100% ultrasound anomalies in deletions (cardiovascular defects, growth restriction) and 50% in duplications. Inherited CNVs (50% deletions, 43% duplications) correlate with milder outcomes, while de novo variants drive high TOP rates (76.5%), emphasizing genomic testing and parental studies for precise counseling.

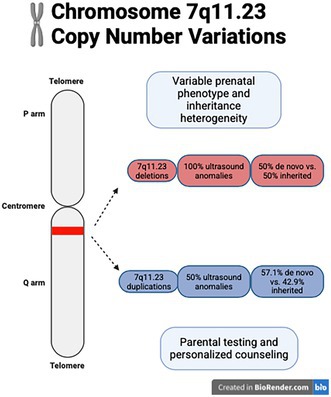

## 1 Introduction

Williams‐Beuren syndrome (WBS; OMIM #194050) is a multisystem disorder caused by a recurrent 1.5–1.8 Mb heterozygous deletion at chromosome 7q11.23. The syndrome is characterized by distinctive clinical features, including cardiovascular anomalies, intellectual disability, and a recognizable facial phenotype (Pober 2010). While the postnatal manifestations of WBS have been extensively documented, the prenatal presentation of 7q11.23 copy number variations (CNVs)—encompassing both deletions and rare duplications—remains poorly characterized, creating significant challenges for prenatal diagnosis and genetic counseling (Berg et al. 2007; Strømme et al. 2002; Morris and Mervis 2000).

Current literature on prenatal WBS primarily consists of isolated case reports or small case series, with fetal growth restriction (FGR) and congenital cardiovascular defects emerging as the most frequently reported findings (Borrell et al. 2018; Yuan et al. 2020; Lv et al. 2023). However, emerging evidence suggests a broader spectrum of phenotypic variability, including less common associations such as aortic coarctation and persistent left superior vena cava (PLSVC) (Yuan et al. 2020; Lv et al. 2023; Wang et al. 2023). Furthermore, the clinical implications of atypical deletions or duplications outside the classic WBS critical region (WBSCR), as well as their correlation with fetal structural abnormalities, remain insufficiently explored (Alesi et al. 2021).

In this study, we present a cohort of 20 consecutive prenatal cases diagnosed with 7q11.23 CNVs. We comprehensively analyzed the indications for genetic testing, prenatal ultrasound findings, inheritance patterns of the CNVs, and pregnancy outcomes. Our objective was to provide a detailed review of prenatal cases involving 7q11.23 CNVs, with the aim of improving the understanding of this genomic disorder in the context of prenatal diagnosis and facilitating more informed clinical decision‐making.

## 2 Materials and Methods

### 2.1 Study Design and Participants

This retrospective cohort study was conducted at the Prenatal Diagnosis Center of the Maternal and Child Health Hospital of Hubei Province (Wuhan, China). From April 2017 to December 2024, 18,459 prenatal samples were analyzed via SNP array/copy number variation sequencing (CNV‐seq), with 3261 (17.7%) diagnosed as chromosomal abnormalities. The study cohort included fetuses with confirmed 7q11.23 deletions or duplications identified through CNV‐seq or SNP array analysis, irrespective of ultrasound findings. Maternal demographics, gestational age at diagnosis, primary indications for genetic testing, ultrasound findings, comprehensive genetic results, inheritance patterns, and pregnancy outcomes were extracted from medical records. The study was approved by the Institutional Ethics Committee of the Maternal and Child Health Hospital of Hubei Province (Approval No. 230741011). Written informed consent was obtained from all participants.

### 2.2 Genetic Testing Methods

#### 2.2.1 SNP Array

Genomic DNA was extracted from uncultured prenatal samples (amniocytes, chorionic villi) and parental peripheral blood using the QIAamp DNA mini kit (QIAGEN, Germany). SNP array analysis was performed using the CytoScan 750 K Array following manufacturer protocols. Data were analyzed using Affymetrix Chromosome Analysis Suite (ChAS) v3.3, with genomic coordinates mapped to GRCh37/hg19 (Affymetrix Inc 2013).

#### 2.2.2 Copy Number Variation Sequencing (CNVseq)

Genomic DNA (10 ng) was fragmented, ligated with adapters, and amplified to construct sequencing libraries as previously described. Libraries were sequenced on the NextSeq CN500 platform (Illumina, USA), yielding approximately 5 million 45‐bp single‐end reads. Reads were aligned to GRCh37/hg19, and CNVs were identified using in‐house bioinformatics pipelines (Wang et al. 2018; Dong et al. 2016).

## 3 Results

### 3.1 Demographics and Genetic Findings

This study included 20 prenatal cases with 7q11.23 CNVs, comprising 13 deletions and 7 duplications, diagnosed between 2017 and 2024. The incidence of 7q11.23 CNVs was 0.11% (20/18,459) overall and 0.61% (20/3261) among chromosomal abnormalities. The mean maternal age was 28.3 years (range: 17–42 years), with a median gestational age at diagnosis of 21 weeks (range: 8–31 weeks). Genetic testing revealed deletions ranging from 0.98 to 4.1 Mb (median: 1.42 Mb) and duplications spanning 1.42–3.03 Mb (median: 1.58 Mb) (Table 1).

**TABLE 1:** Summary of prenatal cases with 7q11.23 copy number variations (CNVs).

Case	Maternal age (years)	Pregnancy history	GA (weeks)	Indication for genetic test	Ultrasound findings	Sex	Diagnostic method	Genetic results (HGVS)	Size (Mb)	Inheritance	Pregnancy outcome
1	30	G2P0	23	Ultrasound findings	Duodenal obstruction	M	CNV‐seq	seq[GRCh37]7q11.23 (72,720,000–74,140,000) × 1	1.42	De novo	TOP
2	27	G2P0	24	Ultrasound findings, adverse pregnancy history	FGR, PLSVC, elevated umbilical artery S/D ratio	M	CNV‐seq	seq[GRCh37]7q11.23 (72,720,000–74,140,000) × 1	1.42	Maternal	TOP
3	30	G2P1	29	Ultrasound findings	Left ventriculomegaly (13 mm), enlarged posterior fossa (11 mm)	M	CNV‐seq	seq[GRCh37]7q11.23 (75,160,000–76,140,000) × 1	0.98	Maternal	Live birth
4	29	G4P1	23	Family history	FGR	M	CNV‐seq	seq[GRCh37]7q11.23 (72,740,000–74,120,000) × 1	1.38	Maternal	TOP
5	29	G1P0	20	Family history	Increased NT (4.8 mm), increased NF (8.1 mm)	F	SNP array	arr[GRCh37]7q11.23q21.11 (76,007,283–80,123,898) × 1	4.1	Maternal	TOP
6	35	G2P0	18	High‐risk NIPT for 7q11.23 microdeletion	Pleural effusion, peritoneal effusion	M	SNP array	arr[GRCh37]7q11.23 (72,664,089–74,143,240) × 1	1.48	De novo	TOP
7	38	G2P1	21	Ultrasound findings, adverse pregnancy history	Increased NT (3.5 mm)	F	SNP array	arr[GRCh37]7q11.23 (72,723,370–74,154,209) × 1	1.43	De novo	TOP
8	21	G1P0	31	Family history, Ultrasound findings	Aortic stenosis, tricuspid regurgitation	M	SNP array	arr[GRCh37]7q11.23 (72,701,098–74,154,209) × 1	1.45	Maternal	TOP
9	25	G1P0	19	High‐risk NIPT for 7q11.23 microdeletion	VSD	F	SNP array	arr[GRCh37]7q11.23 (72,621,346–74,289,047) × 1	1.67	De novo	TOP
10	30	G2P1	28	Ultrasound findings	Rocker‐bottom feet	M	SNP array	arr[GRCh37]7q11.23 (72,650,120–74,154,209) × 1	1.5	De novo	TOP
11	25	G1P0	29	Ultrasound findings, family history	Interruption of the inferior vena cava, micrognathia	M	CNV‐seq	seq[GRCh37]7q11.23 (72,780,000–74,140,000) × 1	1.36	Maternal	Live birth
12	27	G2P0	9	Fetal demise	Fetal demise	M	CNV‐seq	seq[GRCh37]7q11.23 (72,740,000–74,140,000) × 1	1.4	De novo	Fetal demise
13	27	G1P0	11	Fetal demise	Fetal demise	M	CNV‐seq	seq[GRCh37]7q11.23 (72,720,000–74,140,000) × 1	1.42	N/A	Fetal demise
14	32	G1P0	8	Fetal demise	Fetal demise	F	CNV‐seq	seq[GRCh37]7q11.23 (72,200,000–74,140,000) × 3	1.94	De novo	Fetal demise
15	27	G1P0	24	Ultrasound findings	Cleft palate	F	CNV‐seq	seq[GRCh37]7q11.23 (72,720,000–74,140,000) × 3	1.42	De novo	TOP
16	20	G1P0	20	Family history	No abnormalities on ultrasound	F	CNV‐seq	seq[GRCh37]7q11.23 (72,720,000–74,080,000) × 3, seq[GRCh37]2p23.3p23.2 (26,420,000–28,080,000) × 3	1.36/1.66	Maternal (7q), Maternal (2p)	TOP
17	42	G1P0	21	AMA	No abnormalities on ultrasound	M	CNV‐seq	seq[GRCh37]7q11.23 (72,720,000–74,140,000) × 3	1.42	Paternal	Live birth
18	28	G1P0	23	Ultrasound findings	Polyhydramnios, pulmonary artery crossover	M	SNP array	arr[GRCh37]7q11.23 (72,370,954–75,403,750) × 3	3.03	De novo	TOP
19	29	G2P1	29	Ultrasound findings	Unilateral ventriculomegaly (12 mm)	M	SNP array	arr[GRCh37]7q11.23 (72,608,900–74,184,702) × 3	1.58	De novo	TOP
20	28	G1P0	21	Adverse pregnancy history	No abnormalities on ultrasound	M	CNV‐seq	seq[GRCh37]7q11.23 (72,650,001–74,200,000) × 3, seq[GRCh37]4q12 (55,238,386–55,970,791) × 3	1.55/0.73	Maternal (7q), Paternal (4q)	Live birth

Abbreviations: CNV‐seq, copy number variation sequencing; FGR, fetal growth restriction; G, Gravida; GA, gestational age; NF: Nuchal fold; NIPT, noninvasive prenatal testing; NT, Nuchal translucency; P, para; PLSVC: persistent left superior vena cava; TOP, termination of pregnancy.

Seventeen cases (85%) involved the classic WBSCR (chr7:72,700,000–74,100,000). Among the remaining cases, two exhibited larger deletions or duplications extending towards the centromere (Case 5, 4.4 Mb deletion; Case 18: 3.03 Mb duplication), while one case carried a smaller 0.98 Mb deletion (Case 3). Three cases (Cases 12–14) were diagnosed in the first trimester (8–11 weeks) following ultrasound‐detected fetal demise. The remaining 17 cases underwent invasive prenatal testing due to ultrasound anomalies (*n* = 9), family history (*n* = 4), or high‐risk noninvasive prenatal testing (NIPT) results (*n* = 2).

### 3.2 Phenotypic Spectrum

Ultrasound anomalies were observed in 100% (11/11) of cases with available prenatal imaging (Cases 1–11, Table 2). Cardiovascular defects were the most common anomalies, present in 36.4% (4/11) of cases, including PLSVC (Cases 2), aortic stenosis (Case 8), ventricular septal defect (VSD, Case 9), and interruption of the inferior vena cava (Case 11). Other recurrent phenotypes included fetal growth restriction (FGR 18.2%; 2/11; Cases 2, 4) and increased nuchal translucency (NT) (18.2%; 2/11; Cases 5, 7). Unique structural anomalies such as duodenal obstruction (Case 1), unilateral ventriculomegaly (Case 3), and rocker‐bottom feet (Case 10) were each observed in one case.

**TABLE 2:** Ultrasound abnormalities in prenatal cases with 7q11.23 CNVs.

Abnormality	Deletions (*n* = 11)	Cases (Deletions)	Duplications (*n* = 6)	Cases (Duplications)
Cardiovascular defects	36.4% (4/11)	2, 8, 9, 11	16.7% (1/6)	18
Fetal growth restriction	18.2% (2/11)	2, 4	0% (0/6)	—
Increased nuchal translucency	18.2% (2/11)	5, 7	0% (0/6)	—
Ventriculomegaly	9.1% (1/11)	3	16.7% (1/6)	19
Other anomalies*	27.3% (3/11)	1, 6, 10	16.7% (1/6)	15

* Deletion anomalies: Duodenal obstruction (Case 1), Pleural and peritoneal effusion (Case 6), Rocker‐bottom feet (Case 10). Duplication anomalies: cleft palate (Case 15).

Among duplication cases (Cases 15–20), ultrasound anomalies were observed in 50% (3/6), including cleft palate (Case 15), pulmonary artery crossover (Case 18), and unilateral ventriculomegaly (Case 19).

### 3.3 Inheritance Patterns

Inheritance pattern analysis was available for 19 cases. Deletions arose de novo in 50% (6/12) and were inherited in 50% (6/12) (Cases 2, 3, 4, 5, 8, 11). Among the six maternal transmissions, three mothers (Cases 4, 5, 8) presented with mild ID. Two mothers (Cases 2, 4) had adverse pregnancy histories: Case 2's mother had a prior termination of pregnancy (TOP) due to FGR, and Case 4's mother had a child with developmental delay and ocular problem. Case 11 presented with micrognathia, while Case 3's mother was phenotypically normal.

Duplications arose de novo in 57.1% (4/7) and were inherited in 42.9% (3/7) (Cases 16, 17, 20). Case 16's mother harbored 7q11.23 duplication and a 2p23.3‐p23.2 duplication, presenting with short stature (147 cm), poor speech communication, and strabismus. Case 20's mother had prior TOP due to the absence of cerebellar vermis. Case 17's father was phenotypically normal.

### 3.4 Pregnancy Outcomes

TOP was elected in 76.5% (13/17) of ongoing pregnancies, primarily for de novo CNVs or significant ultrasound anomalies (e.g., aortic stenosis, FGR). Families opted to continue the pregnancy in 23.5% (4/17) of cases, resulting in live‐born newborns, all involving inherited CNVs (Cases 3, 11, 17, 20).

## 4 Discussion

This study presents the largest single‐center prenatal cohort to date characterizing the phenotypic spectrum, inheritance patterns, and outcomes of 7q11.23 CNVs. Our findings expand current understanding of the intrauterine manifestations associated with these genomic alterations and have important implications for prenatal diagnosis and genetic counseling.

The universal presence of ultrasound anomalies (100%) in classic WBSCR deletions confirms the high penetrance of prenatal phenotypes in WBS (Kozel et al. 2021). The predominance of cardiovascular defects (36.4%) in our cohort, including aortic stenosis and PLSVC, reinforces the crucial role of *ELN* (OMIM #130160) haploinsufficiency in vascular development. These observations align with the well‐established mechanism whereby *ELN* deficiency disrupts elastin fiber deposition, leading to arterial wall stiffening and subsequent stenosis (Urbán et al. 2000; Hoareau et al. 2023). The novel identification of prenatal markers, such as interrupted inferior vena cava (Case 11), may reflect broader developmental consequences of elastin pathway dysregulation (Collins II 2013).

The chromatin‐remodeling gene *BAZ1B* (OMIM #605681) emerges as another critical contributor to the prenatal phenotype. Our observation of duodenal obstruction (Case 1) and craniofacial dysmorphism (Case 11) provides clinical evidence supporting *BAZ1B*'s role in neural crest cell migration and differentiation (Lalli et al. 2016; Zanella et al. 2019). These findings suggest that *BAZ1B* haploinsufficiency may disrupt transcriptional networks governing both enteric nervous system development and craniofacial morphogenesis, offering a potential explanation for these structural anomalies.

Our cohort included cases with atypical deletions extending beyond the classic WBSCR. Case 5, for instance, carried a 4.4 Mb deletion extending into the 7q21.11 region and exhibited increased NT (4.8 mm) and nuchal fold thickness (8.1 mm), indicative of severe fetal anomalies. Larger deletions often encompass additional genes, contributing to more severe and variable phenotypes, including pronounced neurodevelopmental and structural abnormalities (Weiss et al. 2008). Conversely, Case 3 carried a 0.98 Mb deletion and presented with moderate ventriculomegaly and an enlarged posterior fossa, despite the mother being phenotypically normal. This observation aligns with previous studies suggesting that smaller deletions may retain functional copies of some genes, resulting in attenuated clinical manifestations (Berg et al. 2007). The phenotypic variability observed in atypical WBS underscores the importance of precise molecular characterization of CNVs to improve clinical outcome prediction and facilitate informed genetic counseling (Manning and Hudgins 2010).

While postnatal studies reported WBS as predominantly de novo, our cohort included maternal carriers with mild intellectual disability (Cases 4, 5, 8) who transmitted deletions of variable severity, as well as a phenotypically normal mother (Case 3) who passed a 0.98 Mb deletion. These findings highlight the necessity of parental testing, even in the absence of overt parental phenotypes (Bayés et al. 2003).

The high TOP rate for de novo CNVs (76.5%) reflects parental concerns regarding neurodevelopmental risks, despite the limited prenatal predictability of WBS‐associated ID (Morris and Mervis 2000). Conversely, live births in cases with inherited CNVs (Cases 3, 11, 17, 20) suggest that familial variants may confer milder prognoses, emphasizing the need for nuanced risk communication during genetic counseling.

This study is limited by its retrospective design and small sample size. Future studies should expand cohorts to refine phenotype–genotype correlations, particularly for duplications and atypical deletions. Evidence‐based guidelines should be developed for managing pregnancies with inherited CNVs, integrating molecular diagnostics and multidisciplinary counseling approaches.

## 5 Conclusion

This study advances the prenatal understanding of 7q11.23 CNVs by delineating their heterogeneous phenotypes, inheritance patterns, and counseling challenges. The integration of molecular diagnostics into prenatal workflows is critical for accurate diagnosis and risk stratification. These insights underscore the importance of parental testing and personalized counseling to improve family outcomes. Future studies should prioritize long‐term follow‐up and functional validation to further elucidate the developmental consequences of these genomic alterations and optimize clinical management strategies.

## Author Contributions

**Jiong Yan:** conceptualization, supervision, writing – review and editing. **Ziyang Liu:** formal analysis, writing – original draft. **Song Yi:** investigation. **Nian Liu:** methodology, validation, writing – review and editing.

## Funding

The authors have nothing to report.

## Conflicts of Interest

The authors declare no conflicts of interest.

## Data Availability

The data that support the findings of this study are available on request from the corresponding author. The data are not publicly available due to privacy or ethical restrictions.
